# Experiences of Social Isolation in Older Adults with Chronic Diseases: A Qualitative Study Based on Patients’ Perspectives

**DOI:** 10.3390/healthcare13080902

**Published:** 2025-04-14

**Authors:** Yuqin Jiang, Zhongsu Shi, Annuo Liu

**Affiliations:** School of Nursing, Anhui Medical University, Hefei 230032, China; jiangyuqin@stu.ahmu.edu.cn (Y.J.); shizhongsu@stu.ahmu.edu.cn (Z.S.)

**Keywords:** social isolation, chronic disease, older adults, qualitative research

## Abstract

**Objective:** This study aimed to understand the causes of social isolation among older adults with chronic diseases. **Methods:** In-depth semi-structured interviews were conducted with 20 older adults with chronic diseases, and thematic and interpretative phenomenological analyses were used in order to fully understand the psychosocial and behavioral changes following diagnosis. **Results:** People with severe social isolation described their life journeys after falling ill, and potential influencing factors for social isolation were identified. We identified three themes across biology, psychology, and sociology: declining and imbalanced physiology, negative psychosocial feelings, and unmet relational needs. The biological aspect includes symptom distress, functional disorder, and treatment confinement. The psychological aspect includes emotional stress, feelings of guilt, and stigmatization. The sociological aspect includes meaningless socialization, a lack of confiding relationships, a lack of social support, and the deterioration of the social environment. **Conclusions:** Better identifying the factors and needs that affect social isolation among this population will be invaluable for healthcare professionals and researchers.

## 1. Introduction

Berkman [1] was the first scholar to propose the term social isolation (SI), defining it as the irreversible loss of social emotions and networks. The phenomenon of social isolation is widespread worldwide and has far-reaching health consequences, making it an important risk factor affecting the health of the elderly [2]. Holt-Lunstad [3] believes that the scale of social isolation continues to expand over time, and its impact on the individual mortality risk to a certain extent exceeds that of recognized risk factors such as smoking and obesity. At the same time, it significantly increases the economic burden of healthcare and has become an important public health issue that needs to be addressed.

With the shift in the disease spectrum, chronic non-communicable diseases (CND) have become the leading cause of death and disability worldwide, and their burden is expected to continue to increase [4]. An annual review of chronic diseases shows that about one-third of all adults worldwide suffer from multiple chronic diseases [5]. Given that chronic diseases are more prevalent in older adults, this population is vulnerable to the effects of social isolation during the development of the disease [6,7]. Christiansen’s [8] five-year follow-up survey of older adults with chronic illnesses also confirmed this finding, with older adults affected by long-term chronic illnesses more likely to feel lonely and socially isolated than healthy older adults.

Social isolation has been described as an epidemic among elderly chronic disease patients [9]. Increasing research data show that the incidence of social isolation among elderly people with chronic diseases is relatively high. Meek [10] found, through a survey of chronic disease patients, that 34.5% of elderly chronic disease patients experience social isolation. A previous cross-sectional survey of China’s elderly chronic disease patients revealed that the prevalence of social isolation among this demographic reached 41.2%, a rate significantly above the global average [11]. Elderly chronic disease patients in social isolation are more susceptible to harm compared to healthy individuals [12]. There is increasing evidence that social isolation is associated with negative health outcomes of chronic diseases, such as depression [13], decreased medication adherence [11], and increased mortality [3]. However, the significance of this issue remains underappreciated in China, with screening and intervention strategies to address social isolation in elderly patients with chronic diseases not yet implemented in clinical or community settings [14].

Despite the proposition of a bidirectional causal relationship between chronic illness and social isolation, and the notion that deteriorating chronic health conditions may compel older adults to disengage from social networks, the mechanisms by which social isolation manifests in this specific group of chronically ill older adults remain inadequately delineated [1,15]. Older people with chronic illnesses are a vulnerable group, and their illness experiences may trigger complex psychosocial responses, yet we still know little about this [16]. A significant proportion of the extant literature on social isolation is quantitative in nature, employing scales that merely measure the frequency and extent of social isolation [8,11]. Qualitative research, on the other hand, can be utilized to explore the underlying causes of complex social phenomena, thereby facilitating a more comprehensive and nuanced understanding [4].

Therefore, this study aims to explore how the social health problem of social isolation in elderly patients with chronic diseases occurs through qualitative research combined with China’s sociocultural context, seeking to gain a deeper understanding of patients’ subjective experiences. It also aims to provide a theoretical basis for the subsequent development of social-isolation-related assessment tools for this population and the formulation of measures to prevent and reduce the occurrence of social isolation among elderly people with chronic diseases, in order to prevent the occurrence and development of social isolation at the source.

## 2. Method

An interview guide (Table 1) was constructed based on the conceptual model of social isolation [17]. The five dimensions of this model—the quantity, structure, and quality of social networks and the assessment of relationships related to emotions and resources—comprehensively summarize key measures of social isolation and provide precise theoretical guidance for the identification of characteristics associated with social isolation. Additionally, the biopsychosocial model [18] was employed to elucidate the impacts of illness on social interactions and subjective experiences related to social isolation in older chronically ill patients in terms of physical, psychological, and social dimensions.

### 2.1. Study Design

We conducted semi-structured in-depth interviews using a qualitative descriptive design. The Consolidation of Reports of Qualitative Research (COREQ) standard was also used to ensure the transparency and rigor of the findings [19].

### 2.2. Participants and Setting

By using purposive sampling, we searched for subjects with shared experiences but varied characteristics, such as sex, time since diagnosis, and personal experience, ensuring the sample’s representativeness [20]. We recruited elderly chronic disease patients with social isolation (Lubben Social Network Scale score < 12) from the endocrinology, rehabilitation, oncology, respiratory, cardiovascular, neurology, and other departments of a tertiary hospital in Anhui Province from December 2023 to February 2024. The inclusion and exclusion criteria for the participants are shown in Table 2.

The sample consisted of the elderly with chronic diseases. When analyzing the data, if no new themes were presented, the saturation of the data was considered to have been achieved [21]. When the 17th participant had completed their interview, there was an overlap in information, and three more were subsequently interviewed, with no new information yielded, so the final number was 20; they were numbered N1 to N20. The demographic characteristics and disease information of the participants are shown in Table 3.

### 2.3. Data Collection

The researchers visited the ward, participated in the daily nursing and treatment of elderly patients with chronic diseases, and established a trusting relationship with the patients in the clinical practice. They strengthened their daily communication with patients and their families. In terms of language, the local dialect was utilized to facilitate a rapid rapport with the elderly, to mitigate their guardedness through communication, and to obtain more realistic and emotionally authentic information. This laid a good interpersonal relationship foundation for the later interviews.

The purpose, significance, and method of the study were introduced to the patients and their families, an informed consent form was signed, and the interviews were formally conducted. The interview guide was developed based on a conceptual model of social isolation, using open-ended questions to understand how the participants’ social networks and lifestyles had changed since becoming ill, how much support they had received from different network members, and the forms of this support. We also sought to explore the impact of chronic illness on the structure and functioning of social relationships among older adults. One-on-one interviews were conducted in a separate room of the ward to ensure that the environment was quiet and comfortable, not disturbed by other unrelated personnel, and the privacy of the subjects was protected.

During the interview, the whole process was recorded, including the interviewees’ non-verbal data, such as expressions, movements, and emotions, as well as notes containing the interviewees’ feelings, so as to capture the real situation of the interview to the greatest extent possible when transcribing the text. The researchers remained neutral at all times, listened attentively, asked questions promptly, and avoided inducing language and attitudes. The duration of each interview was controlled at 30 to 40 min.

### 2.4. Data Analysis

In this study, the qualitative research analysis software Nvivo 12.0 was utilized to manage and facilitate data analysis. The utilization of thematic analysis (TA) [22] facilitated the expeditious identification of overarching themes within the data. Interpretive phenomenological analysis (IPA) [23] was employed to comprehend the construction of meaning in individual experiences. This methodological approach, informed by interpretive analysis and the mining of latent meaning, enables the exploration of the mechanisms underlying complex phenomena. The amalgamation of these two approaches enables research that is both comprehensive and in-depth.

All interviews were audio-recorded, with details such as intonation retained when transcribed into textual data. The text was meticulously reviewed to ascertain initial impressions. The data were then subjected to an open coding procedure, during which they were meticulously labeled sentence by sentence and paragraph by paragraph. Short tags, or codes, were used to describe meaningful content, and these codes were subsequently grouped into themes. The original text was revisited, and the coding was double-checked and revised iteratively to refine the high-level themes and form a thematic hierarchy to explain the nature of the phenomenon. To ascertain the consensus of the researchers and confirm the authenticity of the results, a multivariate cross-validation approach was employed. This approach entailed the provision of timely feedback to the participants through participant testing, ensuring that the results accurately reflected their thoughts and experiences through member verification. The data were extracted and analyzed independently by two researchers, and the final results were discussed and reviewed by members of the research team.

### 2.5. Ethical Considerations

The study protocol was approved by the Anhui Medical university ethics committee with the consent (Approval Number: 83242314) and support of the relevant hospital departments. During the course of the study, the purpose, significance, method, confidentiality, and informed consent principles were explained to the interviewees, emphasizing that the patients had the right to withdraw from the study at any time. Informed consent was given after the patients agreed to participate in the study. All participants’ information was kept strictly confidential during the study, and the data supporting the research results were stored at Anhui Medical University.

## 3. Results

**Figure 1:**
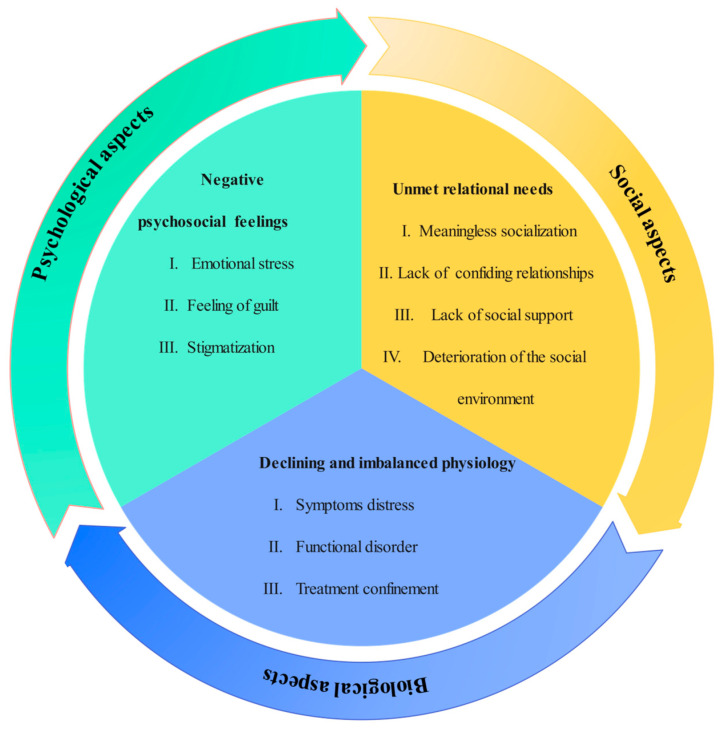
Themes and subthemes based on biopsychosocial modeling.

In this study, elderly people with chronic diseases who were socially isolated described the physiological challenges brought about by the disease, their psychosocial responses after the illness, and their unmet relational needs during their illness. By analyzing the transcripts of the interviews, a series of meaningful themes and sub-themes were developed from the statements of the interviewees. These were summarized into three main themes: declining and imbalanced physiology, negative psychosocial feelings, and unmet relational needs. The biological aspect included three subthemes: symptom distress, functional disorder, and treatment confinement. Three subthemes were related to psychological aspects: emotional stress, feelings of guilt, and stigmatization. Meaningless socialization, a lack of confiding relationships, a lack of social support, and the deterioration of the social environment were the four subthemes of the sociological aspect. The themes and subthemes distilled based on the biopsychosocial model are shown in Figure 1.

### 3.1. Biological Aspects: Declining and Imbalanced Physiology

Topic 1, “Declining and imbalanced physiology”, captures the disease-related factors that caused the participants to experience social isolation, including chronic disease-related symptom distress and dysfunction and treatment confinement. These factors affected the patients’ ability to develop and maintain social relationships.

#### 3.1.1. Symptom Distress

As the disease process accelerated, most participants reported having experienced severe symptoms that severely affected their health and their lives. For example, the symptoms of intermittent sudden seizures forced them to abandon their interests.

N9: “*I used to love exercising and often ran, but now when I suddenly have an illness, I feel chest tightness and can’t catch my breath. Walking is tiring, and after walking for a while, I start panting heavily, making it difficult to go anywhere*”.

N1: “*In 2 years, because of physical discomfort and hipbone pain, I have not danced square dancing. Before, I could exercise with them in the community*”.

#### 3.1.2. Functional Disorder

Some participants indicated that their chronic disease impaired one or even several basic physiological functions, and this severely affected their ability to socially interact and participate in their daily lives.

N19: “*Before suffering from a cerebral infarction, I didn’t hesitate to speak at all. Now, when I say the first sentence, I forget what to say next, and I don’t like to talk to people anymore. So I speak less*”.

N7: “*After getting sick (cerebral infarction), I feel tired and unable to speak clearly. After the sequelae, the ears cannot hear clearly and there is no way to communicate normally with others (sobbing, shedding tears)*”.

N11: “*Because of this disease (diabetes), my legs often hurt, my body is weak, and I can’t walk. My legs are not good and I can’t go anywhere without anyone to take me. I can’t go to visit relatives, so I have to stay at home*”.

A minority felt that the disease did not hinder their ability to perform activities of daily living.

N18: “*I don’t focus too much on the disease and continue to maintain some hobbies (e.g., calligraphy, flower gardening) in my daily life*”.

#### 3.1.3. Treatment Confinement

The participants reported that their individual treatment plans for their chronic diseases included diet and medication requirements and required strict self-management, which forced them to give up the opportunity to participate in some social activities.

N2: “*Because diabetes diet has many taboos, such as no drinking at parties, I usually do not eat with others*”.

N2: “*The insulin pen melted as soon as the temperature rose, so my friends invited me out to play in the summer, and I had to refuse their invitation*”.

### 3.2. Psychological Aspects: Negative Psychosocial Feelings

Topic 2 reveals the real emotions and psychological feelings of the participants after the illness. These negative emotions lead to negative thinking and behavior related to social isolation, and maladaptive social adaptation exposes such individuals to serious social isolation.

#### 3.2.1. Emotional Stress

Many participants recalled the various stages of their illness, claiming that they suffered great psychological stress and that negative emotions such as anxiety, sadness, fear, and loneliness accompanied them through this dark time.

N7: “*I am very sad and distressed, having such a strange illness. Looking at it again, I can’t see it well. Oh, I’ve been feeling so sad all day long. I’m so anxious about this illness that I can only watch TV at home*”.

N17: “*When I first contracted this disease (chronic obstructive pulmonary disease), I often had shortness of breath around two or three in the morning. I was afraid that if I didn’t catch my breath, I would die. I couldn’t sleep at all*”.

N3: “*I live alone in my hometown, and they (children) don’t usually go home. If I suddenly fall ill, no one will know. I feel really lonely*”.

Some participants did not feel that the illness caused them too many negative emotions or had found ways to adapt.

N8: “*Everyone thinks differently when they are sick, some are cheerful and some are not, just face the reality. Since I’m already sick, I’m actively cooperating with the doctor’s treatment. For the next few days, I just want to be comfortable and happy. A good mind is good for good health*”.

#### 3.2.2. Feelings of Guilt

Some participants believed that their illness dragged down their families and imposed a heavy financial and care burden.

N19: “*My life was extended with money, if I hadn’t spent so much money on treatment, I might have died. I hate myself for getting this disease and wasting so much money*”.

N11: “*I think every day that if my illness cannot be cured, I will end my life and not burden my child*”.

N5: “*My husband has been taking care of me for so many years, and I feel guilty for letting him suffer*”.

#### 3.2.3. Stigmatization

Most participants described a significant change in physical image during their illness, as well as stigma that reduced their self-esteem. Aggravating the participants’ disease-related stigma, they chose to self-isolate to prevent interaction with others.

N2: “*The psychological strike is very big, with shadows. I am disabled and different from regular people. When I took a shower or swim, people said that this person was missing a toe*”.

N3: “*I am tortured by illness that my image looks bad. I don’t want people to see me sick, I feel stigmatized*”.

N11: “*Because of this illness (Parkinson’s), when I visited people’s homes, they were afraid of me and dared not let me sit at home. The villagers said that I used to be a capable farmer, but now I can’t even eat. Their discussion hurt me (hurt my self-esteem), and now I don’t want to go out and meet people*”.

### 3.3. Sociological Aspects: Unmet Relational Needs

Topic 3 revealed information about the participants’ social factors related to their experiences of social isolation. After the illness, the changes in their social network structure and interpersonal relationships led to social isolation.

#### 3.3.1. Meaningless Socialization

The majority of the participants who experienced a change in their social mindset due to poor health and reduced social competence reported that they did not want to socialize with people after becoming sick and that they decided to narrow their social networks, especially their networks of friends.

N16: “*I used to be a very enthusiastic person who liked to make friends and fill up my cell phone with contacts. However, I come to realize that the pursuit of such a substantial number of interpersonal relationships is, in fact, an unproductive endeavor. At this stage of life, it is time to “subtract” from my relationships. Being sick made me feel overwhelmed and unwilling to participate those social activities*”.

N12: “*Thinking of terminal disease, I don’t want to socialize with anyone anymore. I’ve lost interest and hope*”.

However, there were a few participants who did not described a narrowed social network.

N15: “*It’s impossible to completely abstain from socializing with people, and keeping myself at home every day isn’t good for my health either. I want to make some more doctor friends around future healthcare needs*”.

#### 3.3.2. Lack of Confiding Relationships

The results showed that the majority of the participants processed the adverse events and emotions experienced during the illness alone. The lack of interaction could be explained by the lack of social connections with older people with chronic diseases with whom to discuss their illnesses, worries, or feelings; it was also related to a lack of specific important relationships.

N8: “*I don’t have any worries, even if I do, I won’t tell anyone. I just need to know for myself. It’s no use telling others about something, no one can help me. I digested my emotions and addressed my concerns on my own*”.

N16: “*There is a big difference in personality between me and my spouse. He doesn’t understand what I say to him, and I want to talk to him but things can’t be resolved. I also don’t have a close relationship with my son and granddaughter, so I don’t want to talk to them about my worries*”.

N14: “*I won’t proactively go to the doctor to discuss my condition because I feel our relationship isn’t that close*”.

#### 3.3.3. Lack of Social Support

Almost all participants indicated a lack of emotional support, such as care and companionship, from network members, and the necessary medical resources and health information for the treatment of their chronic diseases were not provided. The participants claimed that their needs related to medical services were the most urgent, highlighting the convenience of the medical treatment process, the promotion of door-to-door medical services, the accessibility of medical resources, the need for health knowledge, access to health information, and the popularization of healthcare policies.

N5: “*My son doesn’t see me all year round and doesn’t care about me. He has several children to raise at home, and now it’s difficult to earn money. He can’t even take care of himself, how can he take care of me*”.

N3: “*For the five years before falling ill, it was my son who paid for medical expenses and brought me to see a doctor. This year, my son doesn’t care about me anymore. He hasn’t called me and even hit and scolded me. My life is tough*”.

N1: “*No one helped me pay for medical treatment. I did it myself. My daughter drove me to hospital, but I took care of all the medical treatment myself*”.

N19: “*Medical expenses are high, and the money for medical treatment is borrowed from relatives and friends. Some relatives and friends are unwilling to keep in touch with us because we can’t repay the borrowed money timely*”.

N1: “*I went to the hospital for a check-up because I didn’t understand anything. I asked questions from upstairs and downstairs, running around and feeling a lot of pain all over my body. I hope the number of inspection items can be reduced, and the inspection sites can be located together. If the community can increase on-site medical services, there will be no need to run around*”.

#### 3.3.4. Deterioration of the Social Environment

Some participants claimed that they needed disease treatment or moved in with their children. After leaving their own communities, the stable social network structure is broken. They are faced with new problems, such as poor environmental adaptation, and experience dilemmas related to social integration.

N13: “*After retirement, I sold my house and moved here to help my daughter with my grandson. Before I moved here, I had some old colleagues and friends. Before moving here, I had contact with some old colleagues and friends. After moving here, I didn’t know anyone here anymore, and taking care of my grandson has already taken up most of my time*”.

N6: “*I came here from my hometown to help take care of my grandson. I didn’t go back all year round and contact my relatives and friends in my hometown. After getting sick, I basically stay at home, and do not know anyone*”.

N3: “*After getting sick, I often go out to see a doctor, most of the time in the hospital*”.

## 4. Discussion

The purpose of our study was to understand participants’ experiences in order to explore the mechanisms of social isolation in older people with chronic diseases, which could guide and help researchers in conducting in-depth research on the elderly population and their experiences of social isolation. Through a qualitative analysis, we identified three themes: (1) physiological aspects—declining and imbalanced physiology, (2) psychological aspects—negative emotions and psychosocial feelings, and (3) social aspects—unmet relational needs.

In terms of physiology, our study supports the existing results linking chronic illness with social withdrawal and reduced social activity [24]. The social challenges of symptom distress, dysfunction and treatment limitations associated with physiological decline and imbalance associated with chronic diseases severely impacted the ability of most participants to maintain their lifestyles, physical activity and leisure activities, all of which were closely linked to their social interactions. The different experiences of a small number of participants may have been due to the reduced severity of their diseases. Regarding the psychological aspect, psychological dysregulation, which manifests in negative emotions such as sadness, anxiety, fear, and loneliness, is a prominent phenomenon among elderly individuals affected by chronic diseases [25]. Regarding the limited number of individuals who exhibited an optimal state of mind during the course of their illness, their experiences suggest that a favorable mental state is significantly associated with the promotion of health [26]. The progression of chronic diseases increases the burden on patients and their families, leading to serious psychological impacts, such as self-blame and guilt, in the elderly. In the process of interpersonal communication, due to the fear of rejection and discrimination, negative impacts such as stigma and loneliness are worsened, resulting in the typical characteristics of social isolation—social withdrawal behavior [27]. From the perspective of social psychology, excessive self-attention in elderly people with chronic diseases can easily lead to negative emotional cycles. If it cannot be regulated through external support, it will further affect their cognition and behavior and promote the continuous development of social isolation [28].

Sociological aspects were prominent in this study. Our research found that elderly people with chronic diseases find it difficult to establish confiding relationships with others. However, previous studies have not reported a causal relationship between a lack of communication and social isolation. Our research provides evidence that the lack of confiding relationships in elderly people with chronic diseases may be related to emotional conflicts caused by the disease, leading to relationship breakdowns. It may also be due to differences between the patients’ expectations and the actual support obtained after communicating with others during the progression of the disease, which reduces the patient’s desire to confide in others and leads to self-isolation. Newton [29] found that, in primary healthcare settings, older people without a confidant often experience more severe anxiety and depression. Sedrak [30] found that limited communication with professionals and families hinders access to health information. This prevents patients from seeking health information to help with disease management and medical decision-making, and this information scarcity may have negative consequences. These findings further validate previous studies showing that the absence of confiding relationships has adverse consequences [31]. In addition, a lack of communication with relatives, friends, and healthcare providers is also detrimental to the regulation of patients’ negative emotions, which may lead to social isolation [32]. Healthcare providers, as the only form of regular face-to-face social interaction for many patients, should emphasize early screening for the risk of social isolation among elderly people with chronic diseases and help them to become more aware of the dangers of social isolation through publicity and education. It is necessary to focus on the needs of patients, meet their medical service and health information needs as much as possible, and improve their medical satisfaction.

Meaningless socialization, despite being a secondary theme, was significant in this study. Previous studies have shown that small social networks and low levels of social activity are favorable predictors of social isolation and loneliness in older adults [33]. However, this study found that less social contact related to the disease and age does not equally affect all aspects of people’s social networks. Changes in some patients’ willingness to socialize and adjustments in their social networks provide new perspectives for interpretation. Small but high-quality social networks may be more important for older patients with chronic diseases. They optimize their social networks by eliminating less satisfactory relationships. Adjusting their social networks to adapt to their chronic health conditions provides energy to better cope with and manage their illnesses, which may lead to better outcomes and prognoses. According to socioemotional selection theory, the transformation of social networks in elderly patients with chronic diseases may be related to their perceived limited futures [34]. The need for supportive healthcare networks, while retaining the closest relationships, shifting their social networks to home-based networks, and expanding their connections with healthcare workers, seem more important. As reported by a recent study, this change in network types may stem from the emphasis on “group harmony” and “interdependence” in the traditional Chinese culture of Confucianism, where the elderly are more dependent on blood relationships [35]. The participants’ perspectives on the structure and quality of their social networks provide insights for future intervention programs aimed at preventing social isolation and individualized to help older people with chronic illnesses to build small, high-quality social networks. For patients with shrinking social networks, social workers can help to build platforms to promote active, meaningful social connection with others and provide the resources that they need to prevent social isolation and loneliness, enabling them to better cope with their own diseases.

The experiences of older adults with chronic diseases highlight the need for additional support services and resources to enable them to better cope with their health problems [36]. Moreover, the results indicate a serious lack of social support from relationship networks during their illnesses. This is not conducive to recovery from the disease and may further worsen the health of elderly patients with chronic diseases. Sociologist Xiaotong Fei [37] believes that, differing from the western “relay model”, China’s family model reflects a “feedback model”. Shuo Wen Jie Zi [38] refers to “filial piety”, where children have the obligation to raise and take care of their parents, including material giving, care provision, and spiritual comfort. According to research, those who live with their children lack interaction, which is contrary to the traditional Chinese culture of filial piety [39]. Moreover, with the transformation of the traditional family structure and pension model (family life model and intergenerational coexistence model) in China, it has become difficult for family caregivers of the elderly with chronic diseases to balance their care responsibilities with the pressures of child care. The dual pressure of the economic burden and care burden caused by the disease results in the absence of crucial family support, further aggravating the social isolation of elderly individuals with chronic diseases. Traditional Chinese values are collapsing, such as family cohesion and traditional care and support [40]. Therefore, it is particularly important to restore the culture of filial piety and emphasize the notion of “respecting and loving the elderly” in today’s society. We suggest that family caregivers should provide sufficient care and support to patients so as to enable them to cope with their diseases and address the poor cognition and behavior of such patients.

## 5. Advantages and Limitations

A major strength of the current study is our analysis of the mechanisms underlying the occurrence and development of social isolation in the overall population of older adults with chronic diseases, as well as discussing these associations based on the specific Chinese cultural context, which cannot be found in previous studies. It is crucial to comprehensively analyze the social isolation of elderly people with chronic diseases based on physiological, psychological, and social aspects, because the nature of interpersonal relationships among adults in the context of socioeconomic and cultural development has changed greatly, affecting their capacity to cope with deteriorations in health. Our results highlight the causal relationship between physical, psychological, and social isolation in elderly people with chronic diseases in a specific cultural context. Other strengths of our study are the inclusion of primary healthcare providers in semi-structured interviews with members of patients’ social networks and the consideration of their roles as the sole social contacts of some elderly patients with chronic diseases.

However, some limiting factors need to be considered. Firstly, we interviewed patients during admission for acute episodes, so their responses may not accurately reflect the problems that affect the patients’ daily lives. More studies with larger follow-up periods are needed to ensure a comprehensive perspective on the social isolation of older adults with chronic conditions. Secondly, although we used the largest difference sampling strategy to enrich the participants’ demographic characteristics as much as possible, and the interviews reached data saturation, the themes identified in this study may underestimate the causes of social isolation in larger populations, such as participants not experiencing chronic illnesses, and the disease severity was not considered among the participant characteristics. Thirdly, this study only considered the experiences of social isolation among the elderly with chronic diseases from the perspective of the patients. Finally, our interview guide was not comprehensive and did not address all potential areas where social isolation among chronic patients may be disadvantageous.

## Figures and Tables

**Table 1:** Semi-structured interview guide.

Interview Guide
(1) Since your diagnosis, what impact do you think the disease has had on your life?
(2) Could you please describe your current social interaction situation?
(3) How is your current relationship with the people around you? Who are the people you confide in?
(4) How do you feel the people around you treated you after you fell ill?
(5) Could you talk about what new needs you have for the people around you since you were diagnosed with chronic diseases?

**Table 2:** Inclusion and exclusion criteria for the participants.

Inclusion Criteria	Exclusion Criteria
(1) After clinical diagnosis of chronic disease and ongoing treatment (such as medication, etc.)	(1) Patients with other non-chronic diseases and infectious diseases
(2) Aged 60 years old and above, with clear thinking, able and willing to cooperate with the investigation	(2) Unable to express themselves independently, cognitive impairment or serious illness, unable to cooperate
(3) A Lubben Social Network Scale score of <12 points	(3) Those who interrupted the interviews for various reasons

**Table 3:** Demographic characteristics and disease characteristics.

Variable	Count	Frequency (%)	Mean Value ± Standard Deviation
Age			71 ± 7.14
Sex			
Man	12	60	
Woman	8	40	
Domicile			
Rural area	8	40	
City	12	60	
Dwelling state			
Living alone	4	20	
Living with partner	11	55	
Joint habitation (including children)	5	25	
Marital status			
Married	16	80	
Divorced	3	15	
Bereaved	1	5	
Degree of education			
Elementary school or below	6	30	
Primary school and above	14	70	
Previous career			
Peasant	7	35	
Staff and workers	13	65	
Number of children			
1	8	40	
2	9	45	
3	3	15	
Number of chronic diseases			
1	12	60	
2	7	35	
≥3	1	5	
Time since disease diagnosis (years)			
≤1	3	15	
1~10	12	60	
≥10	5	25	

## Data Availability

The data presented in this study are not available due to privacy.
